# Nefazodone shortage in the United States: analysis of drug switching and utilization

**DOI:** 10.3389/fpsyt.2025.1613275

**Published:** 2025-09-30

**Authors:** Ashley Tabah, Julia Fox, Clayton English

**Affiliations:** The CHOICE Institute, School of Pharmacy, University of Washington, Seattle, WA, United States

**Keywords:** drug shortage, nefazodone, claims analysis, mental health, psychiatry

## Abstract

**Introduction:**

Medication shortages and manufacturer discontinuations of antidepressants place patients in vulnerable scenarios if medications become inaccessible, leaving prescribers to alter therapy without guarantee of a sustained response to the new treatment.

**Methods:**

We conducted a descriptive retrospective analysis to explore changes in drug utilization and switching patterns following an FDA-declared nefazodone shortage in the United States. Using the Komodo Healthcare Map®, we analyzed prescribing patterns across the pre-shortage, shortage, and post-shortage periods.

**Results:**

A total of 7891 individuals were selected for inclusion, with 2185 eligible for the switching outcomes. A majority (96.4%) switched to an alternative medication, with an average time to switch of 106.8 days (SD: 81.7). Few patients resumed nefazodone use post-shortage (7.3%).

**Discussion:**

Although most patients transitioned to alternative antidepressants during the shortage, the need for treatment changes and the low rate of returning to the original medication may reflect challenges to patient-centered care. There is a need for clearer prescriber guidance to better support clinical decision-making during medication shortages.

## 1 Introduction

Effective treatment of major depressive disorder and other mood disorders often necessitates an individualized approach, with antidepressant medications playing a critical role in symptom management and functional stability (1). However, drug shortages or manufacturer discontinuations can disrupt a patient’s treatment, placing them in vulnerable situations and leaving prescribers to find rapid solutions. These disruptions may compel prescribers to switch patients to alternative therapies, a process that carries clinical uncertainty, as the effectiveness and tolerability of a new treatment may vary across individuals (2).

Nefazodone, a second-generation antidepressant, is one of two antidepressants with the unique mechanism of action of blocking both the serotonin transporter and post-synaptic serotonin 2A receptors (3). It is also the only antidepressant carrying a United States Food and Drug Administration (FDA) boxed warning for severe liver toxicity, with an estimated risk of 1/250,000 to 1/300,000 patient-years of treatment, thus its clinical utility is primarily reserved for specialized cases (4). There are several reasons clinicians continue to use nefazodone despite its hepatotoxic risks, including its lower propensity to cause sexual side effects relative to other serotonergic antidepressants and its demonstrated benefit in treating sleep disturbances associated with post-traumatic stress disorder given its minimal impact on disrupting sleep architecture (5–7). Nefazodone is not endorsed as a first-line treatment option and is typically incorporated after failed trials of other treatments (8). Therefore, it is sometimes used for resistant forms of depression, despite a lack of studies demonstrating its benefit in this population.

Nefazodone faced an FDA-declared shortage from September 2020 to June 2022, which allows us to observe prescribing patterns and switching behaviors during a period of constrained availability (9). Medication shortages such as this one highlight broader concerns regarding continuity of care, especially for patients reliant on stable pharmacologic regimens.

In this observational analysis, we aim to describe the prescribing trends and switching behaviors among patients impacted by the nefazodone shortage. Specifically, we examine the extent of drug switching, time-to-switching, and utilization patterns of alternative medications during and after the shortage period. Our research contributes to a limited body of literature on the implications of medication shortages for psychiatric patients, which has often been eclipsed by provider shortages (10–12).

## 2 Methods

### 2.1 Data source

We used the Komodo Healthcare Map^®^ between January 2016 and September 2023, a comprehensive database capturing real-world healthcare data from a nationally representative U.S. population (13). It includes detailed patient demographics, monthly insurance eligibility, medical (inpatient and outpatient) and pharmacy events, and provider information. The platform integrates longitudinal data, enabling analysis of healthcare utilization, treatment patterns, and outcomes over time. Its scope allows for tracking changes in medication use, understanding healthcare delivery, and identifying trends in response to external factors like medication shortages.

### 2.2 Study design

**Figure 1:**
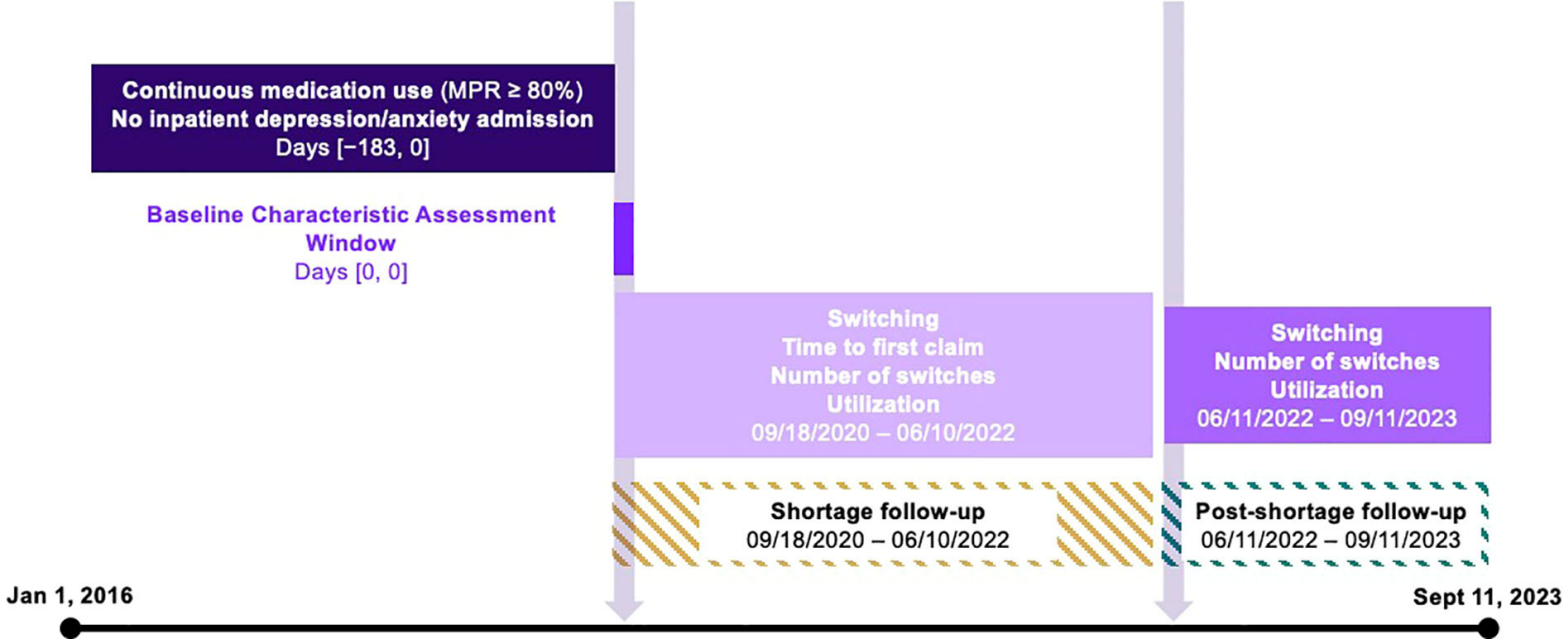
Study design.

Subjects were selected based on their use of nefazodone. The index date (September 18, 2020) was defined by the FDA’s declaration of a shortage for nefazodone. We identified three periods for our analysis: the pre-period, defined as the six months prior to the index date; the shortage period from September 18, 2020 to June 10, 2022; and the post-shortage period from June 11, 2022 until the end of data availability on September 11, 2023 (**Figure 1**). Nefazodone was considered the baseline drug and any different antidepressant used in the shortage- and post-period was considered a new drug.

### 2.3 Sample selection

Our analytic sample included individuals with a prescription claim for nefazodone. We required continuous nefazodone use, as defined by a medication possession ratio (MRP) ≥80%, and no inpatient hospitalization for depression or anxiety in the six-month pre-period. Individuals were excluded if their MPR for other antidepressants in the shortage and post-periods was below 80%. For the switching outcomes, we further restricted the sample to individuals who had a prescription claim for any antidepressant in all three periods.

### 2.4 Analysis and outcomes

The primary outcome was the count of nefazodone claims across all three study periods. Secondary outcomes included the proportion of individuals who switched antidepressants, the average time to switching, the number of switches during the shortage (categorized as 0, 1, 2, or 3+), and the types of alternative antidepressants used.

Switching was defined as the initiation of a new antidepressant during the shortage or post-shortage period, with a medication possession ratio (MPR) of at least 80%. A medication was considered new if there were no claims for it during the six-month pre-index period. Individuals were categorized as having switched even if they also had claims for nefazodone during the shortage or post-shortage periods.

Data processing was performed on the SQL Snowflake platform and the analyses were conducted using R Studio, version 4.2.1 (RStudio, PBC, Boston, MA).

## 3 Results

### 3.1 Sample

A total of 7891 individuals were selected for inclusion, with 2185 eligible for the switching outcomes. The utilization sample had a mean age of 63.0 (standard deviation (SD): 11.8) years, 61.8% were female, and 42.4% were enrolled in Medicare for their prescription drug insurance. Characteristics were similar for the switching sample in which the mean age was 62.9 (SD: 11.4) years, 63.2% were female, and 42.5% were enrolled in Medicare for their prescription drug insurance (**Table 1**).

**Table 1:** Baseline characteristics

Characteristic	Utilization (n=7,891)	Switching (n=2,185)
Age		
Mean (SD)	63.0 (11.8)	62.9 (11.4)
Median	64.7 [11.7, 86.7]	63.7 [11.7, 86.7]
NR	72 (0.9%)	12 (0.5%)
Sex		
Female	4936 (61,8%)	1361 (63.2%)
Male	3044 (38.1%)	823 (37.7%)
NR	1 (0.0%)	1 (0.0%)
RX insurance		
Commercial	3818 (47.8%)	1053 (48.2%)
Medicare	3386 (42.4%)	929 (42.5%)
Medicaid	357 (4.5%)	95 (4.3%)
NR	420 (5.3%)	108 (4.9%)

SD, standard deviation; RX, prescription drug; NR, not reported.

### 3.2 Switching

**Figure 2:**
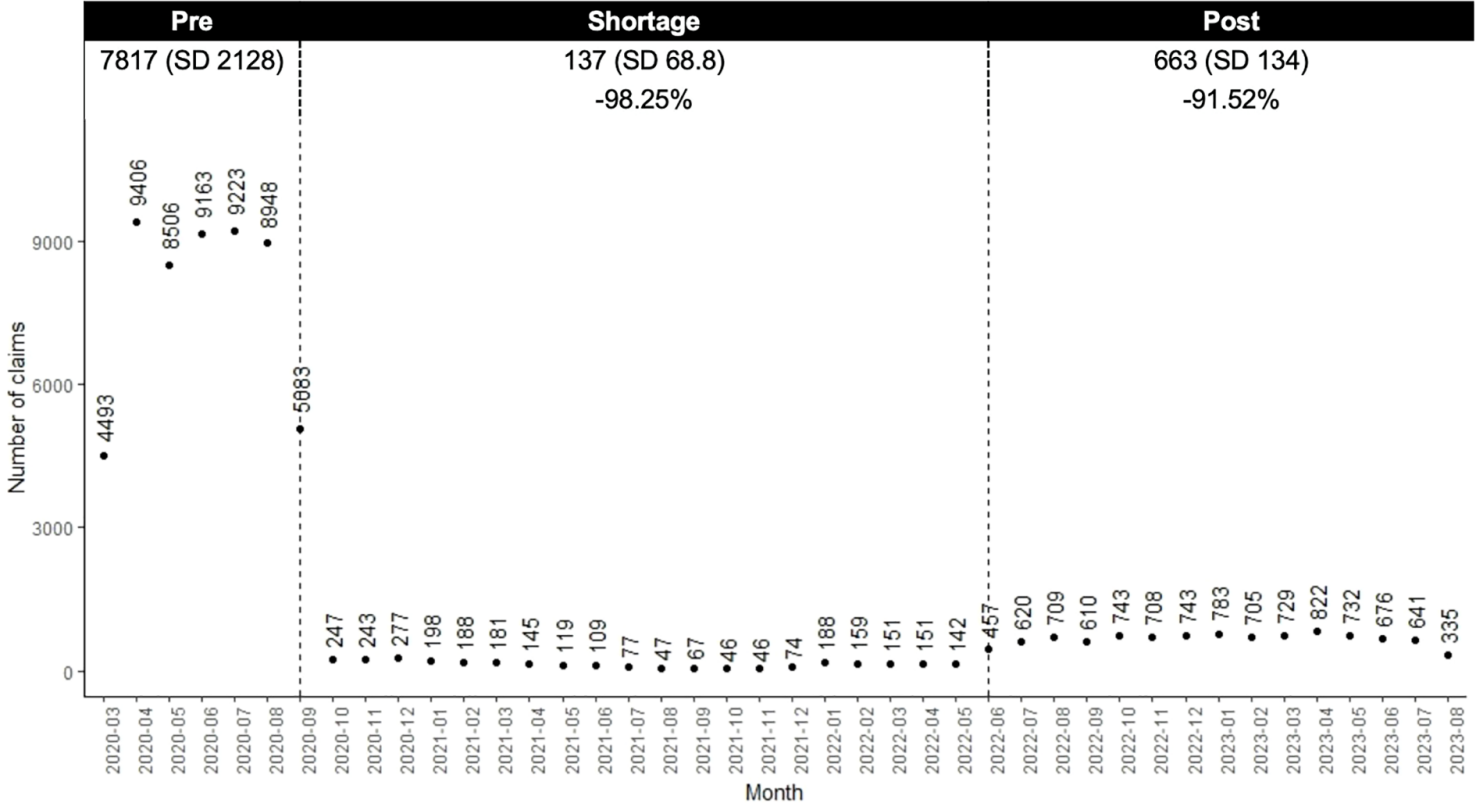
Number of nefazodone claims in each study period.

Compared to the pre-shortage period, the number of nefazodone claims decreased 98.3% during the shortage and by 91.5% post-shortage (**Figure 2**). Within the switching sample, 96.4% (n=2107) of individuals switched from nefazodone to another medication, with an average time to switch of 106.8 days (SD 81.7). Most switchers (n=1941; 92.1%) switched to one new drug, 7.6% (n=159) to two, and 0.3% (n=7) to three or more (**Table 2**). During the shortage and post-period, trazodone was the most common drug initiated (39.1% and 38.8% of claims, respectively).

**Table 2:** Number of drug switches during the shortage and post-shortage periods (n=2,107).

Number of switches	Shortage	Post
1	1941 (92.1%)	1767 (83.9%)
2	159 (7.6%)	273 (13.0%)
3	7 (0.3%)	21 (1.0%)

Denominator n=2107: The number of individuals taking a drug other than nefazodone during the shortage.

## 4 Discussion

We conducted a descriptive retrospective analysis of nefazodone utilization after the FDA declared shortage on September 18, 2020. Our findings showed that the shortage coincided with a change in utilization patterns for nefazodone, with a decrease in nefazodone claims, and a subsequent uptake and utilization of other antidepressants. After cessation of the shortage, nefazodone claims did not return to pre-shortage levels. This study addresses a largely neglected issue within the literature by focusing on the understudied area of psychiatric medication shortages and their impact on treatment patterns, providing practical information for both clinicians and policymakers.

Nefazodone is one of several second-generation antidepressants available; however, its unique mechanism of action is a barrier to finding an easily convertible and interchangeable equivalent. Given its utilization for specialized cases, we anticipated claims to rebound after conclusion of the shortage; however, there was only a small return in utilization. Most patients in our sample switched to alternative medications with minimal return to nefazodone, which may reflect the extended duration of the shortage. The length of the shortage may have allowed time for patients to adjust to their new medication, made prescribers hesitant to prescribe in fear of another shortage, or resulted in a lack of awareness that the shortage had ended. However, such adaptations may not always mitigate the immediate risks of disrupted care or reflect potential adverse effects of switching, emphasizing the need for continued research in psychiatric medication shortages.

We found that trazodone was the most common drug initiated during and after the shortage. This is likely because trazodone is the closest structurally and mechanistically equivalent therapy; however, it is largely used as a hypnotic in clinical practice secondary to its potent histamine antagonism (14). We hypothesized that mirtazapine would also be a common treatment alternative given it possesses some pharmacological overlap in blocking serotonin receptors; however, its utilization was less than other common antidepressants (3.9% during shortage), including two selective-serotonin reuptake inhibitors (i.e., escitalopram [10.6%], sertraline [7.4%]). Apart from trazodone, antidepressants initiated during the shortage aligned with first-line therapies recommended by the Canadian Network for Mood and Anxiety Treatments and the American Psychiatric Association guidelines, including selective serotonin reuptake inhibitors, serotonin-norepinephrine reuptake inhibitors, and bupropion (8, 15). While our study does not assess clinical outcomes, the fact that most patients did not return to nefazodone following the shortage may reflect, in select cases, a transition to safer or more guideline-aligned treatments. Given nefazodone’s boxed warning for hepatotoxicity, it is possible that the shortage prompted prescribers and patients to consider alternative options they may not have pursued otherwise.

Our study’s focus on descriptive and quantifiable outcomes, such as time-to-switch and frequency of switches, offers an important foundation for understanding the impact of psychiatric medication shortages, an area where limited research exists. While prior studies have examined shortages in oncology and antimicrobial therapies, with many studies demonstrating significant negative, clinical, economic, and humanistic consequences, these findings may not be directly applicable to chronic mental health conditions (16). Many chemotherapeutics and antimicrobials are used for time-limited treatment courses, whereas psychiatric medications are often prescribed for long-term, continuous use, especially for treatment-resistant indications. Our study contributes to closing this gap for psychiatric medications by using nefazodone as a case study. Nefazodone use is largely limited to a small patient population for specific clinical purposes and therefore can be easily tracked in claims data compared to drug classes like psychostimulants, which have multiple therapeutic and generic alternatives within the class. Although the niche use of nefazodone enhances our ability to study switching patterns, these findings reflect a specialized context that could differ from experiences with other psychiatric medications or with shortages that are limited in time duration. Nonetheless, our work establishes a needed starting point for evaluating the broader impact of psychiatric medication shortages. While impacts across medication classes may be different, it is important to further investigate if psychiatric medication shortages have a similar impact compared to other therapeutic areas.

This research also underlines the critical need for evidence-based guidelines to support prescribers during shortages as they navigate sudden changes in available options for patient care. Shortages complicate the already complex process of tailoring treatment to individual patient needs, creating uncertainty in how best to proceed. Our findings suggest that intentional, patient-centered treatment decisions may be disrupted by supply chain issues. While our data did not capture direct patient or prescriber perspectives, these disruptions are likely to create challenges in practice. To address these challenges, future research should prioritize understanding patient and prescriber perspectives through mixed-methods approaches and assessing the long-term effects of treatment changes on patient outcomes.

### 4.1 Strengths

This research has various strengths, including the potential generalizability of our findings to patients prescribed nefazodone. The use of the Komodo Healthcare Map^®^, a large, nationally representative claims database, captures insurance claims data from a socioeconomically diverse sample of over 330 million adults in the United States. Additionally, the use of the ≥80% MPR threshold aligns with established standards in adherence research and ensures robustness in classifying treatment transitions.

### 4.2 Limitations

Our study also faces some important limitations that must be considered when interpreting the results. First, given that this is a descriptive analysis, we are unable to establish causation. Therefore, we cannot be sure that the utilization and switching we observe are due to the shortage and not to some other factors. Second, our definition of switching included individuals who had overlapping claims for nefazodone and a new antidepressant. While this approach aimed to capture the complexity of prescribing behaviors during a shortage period, it may have included individuals who were not true switchers or who resumed nefazodone use later. As such, this may have introduced some misclassification and resulted in an overestimation of our switching estimates. Third, trazodone is used clinically as an antidepressant and a sleep aid. Therefore, while trazodone was the most commonly initiated antidepressant during the shortage period, we could not determine the indication for which it was dispensed. In fact, nefazodone has been used off-label for sleep disturbances, particularly in patients with post-traumatic stress disorder (17). As such, it remains uncertain whether the observed switching accurately reflects treatment for insomnia, depression, or both. Fourth, the nature of claims data is in itself a limitation due to potential coding errors and data misclassification. Additionally, the lack of data on clinical outcomes, such as treatment effectiveness, tolerability, safety, or quality of life, which all may have contributed to medication switching, cannot be evaluated. Moreover, findings cannot be generalized to uninsured and all Medicare populations. Finally, unmeasured prescriber-level factors (e.g., formulary requirements) may influence prescribing decisions and influence our results regardless of a scarcity in supply in medication. These limitations underscore the need for future research to explore the broader patient impacts of medication shortages.

## 5 Conclusion

In this descriptive analysis, the FDA-declared nefazodone shortage was followed by a decrease in the number of nefazodone prescription drug claims, an increase in the claims for other drugs, mainly trazodone, and an average time to drug switching of just over three months. This study can serve as a foundation for further exploration of psychiatric medication shortages and their impacts on patient outcomes. Such research will be essential to developing strategies and guidelines to ensure continuity of care and minimize the impact of shortages on vulnerable patient populations.

## Data Availability

The data analyzed in this study is subject to the following licenses/restrictions: The datasets analyzed for this study can be found in the Komodo Healthcare Map^®^. Requests to access these datasets should be directed to https://www.komodohealth.com/solutions/healthcare-map/.
